# Low-molecular-weight heparin therapy reduces 28-day mortality in patients with sepsis-3 by improving inflammation and coagulopathy

**DOI:** 10.3389/fmed.2023.1157775

**Published:** 2023-06-09

**Authors:** Ze Zhang, Taotao Yan, Danfeng Ren, Jingwen Zhou, Liangru Liu, Juan Li, Shan Fu, Tianzhi Ni, Weicheng Xu, Yuan Yang, Tianyan Chen, Yingli He, Yingren Zhao, Jinfeng Liu

**Affiliations:** ^1^The First Affiliated Hospital, Xi'an Jiaotong University, Xi'an, China; ^2^Shaanxi Clinical Medical Research Center of Infectious Diseases, Xi'an, China; ^3^Institution of Hepatology, The First Affiliated Hospital, Xi'an Jiaotong University, Xi'an, China

**Keywords:** low-molecular-weight heparin, disseminated intravascular coagulation, sepsis, mortality, propensity score

## Abstract

**Background and aim:**

Sepsis is a syndromic response to infection and is associated with high mortality, thus imposing a significant global burden of disease. Although low-molecular-weight heparin (LMWH) has been recommended to prevent venous thromboembolism, its anticoagulant and anti-inflammatory effects in sepsis remain controversial. Owing to the modification of the Sepsis-3 definition and diagnostic criteria, further evaluation of the efficacy and benefit population of LMWH is required.

**Methods:**

We performed a retrospective cohort study to assess whether LMWH improved the inflammation, coagulopathy, and clinical outcomes against Sepsis-3 and to identify the target patients. All patients diagnosed with sepsis at the First Affiliated Hospital of Xi'an Jiaotong University (the largest general hospital in northwest China) from January 2016 to December 2020 were recruited and re-evaluated using Sepsis-3 criteria.

**Results:**

After 1:1 propensity score matching, 88 pairs of patients were categorized into the treatment and control groups based on subcutaneous LMWH administration. Compared with the control group, a significantly lower 28-day mortality was observed in the LMWH group (26.1 vs. 42.0%, *p* = 0.026) with a comparable incidence of major bleeding events (6.8 vs. 8.0%, *p* = 0.773). Cox regression analysis showed that LMWH administration was the independent protective factor for septic patients (aHR, 0.48; 95% CI, 0.29–0.81; *p* = 0.006). Correspondingly, the LMWH treatment group showed a significant improvement in inflammation and coagulopathy. Further subgroup analysis showed that LMWH therapy was associated with favorable outcomes in patients younger than 60 years and diagnosed with sepsis-induced coagulopathy (SIC), ISTH overt DIC, non-septic shock, or non-diabetics and in patients included in the moderate-risk group (APACHE II score 20–35 or SOFA score 8–12).

**Conclusion:**

Our study results showed that LMWH improves 28-day mortality by improving inflammatory response and coagulopathy in patients meeting Sepsis-3 criteria. The SIC and ISTH overt DIC scoring systems can better identify septic patients who are likely to benefit more from LMWH administration.

## Introduction

Sepsis, with an estimated number of cases between 18 and 31.5 million per year and representing ~20% of all global deaths, remains one of the leading health burdens worldwide and is associated with high healthcare costs (1, 2). Increasing evidence indicates (3–6) that extensive coagulopathy develops due to uncontrolled inflammatory responses and endothelium injury, which is speculated to contribute to the pathogenesis of multi-organ dysfunction syndrome (MODS), disseminated intravascular coagulation (DIC), and even death. Depending on the pathogenesis of sepsis, anticoagulants can be theoretically used as a potential therapeutic agent for treating sepsis. However, the efficacy of anticoagulant therapies in sepsis continues to remain a controversial issue. Recently, anticoagulant therapy with low-molecular-height heparin (LMWH) and unfractionated heparin has been extensively recommended for non-critically ill patients with COVID-19, owing to their antithrombotic and anti-inflammatory properties (7, 8). Hence, it is necessary to reevaluate the clinical efficacy of anticoagulant therapy in sepsis.

Heparin exhibits anticoagulant properties by primarily enhancing the activity of antithrombin and causing the inactivation of factors Xa and thrombin (IIa). It is also known to inactivate other coagulation factors, such as FXIIa and FXIa, depending on the type of heparin administered (6), although to a lesser extent. During the manifestation of sepsis, thrombin generation is closely associated with inflammation. Heparin thus acts as an anti-inflammatory agent and was first applied in the treatment of sepsis in 1966 (9). Initially, unfractionated heparin, guided by activated partial thromboplastin time, particularly when administered intravenously, was recommended for the prevention of venous thromboembolism in non-surgical patients with sepsis (10). Owing to the smaller molecular weight of LMWH compared to unfractionated heparin, the administration of LMWH reduced the incidence of heparin-induced thrombocytopenia and the risk of bleeding (11). Therefore, LMWH is considered to be a more effective and safe anticoagulant option. A recent meta-analysis based on randomized controlled trials demonstrated the effectiveness of LMWH in improving coagulation dysfunction and inflammatory response and reducing the incidence of MODS and 28-day mortality (12). However, various other clinical trials evaluating the role of anticoagulants, including LMWH, did not report significant benefits for this compound (13–16). These discrepancies can be attributed to the heterogeneity of the enrolled population, including diverse causative factors, the timing of treatment, and the dosage of heparin.

Inflammation and coagulation are inextricably linked to the progression of sepsis. DIC, the severe form of coagulopathy, can induce organ dysfunction and lead to higher mortality. Although there is no uniform standard definition and diagnostic criteria for DIC, the International Society on Thrombosis and Haemostasis (ISTH) overt DIC scoring system (17), the Japanese Association for Acute Medicine (JAAM) DIC scoring system (18), and the Chinese DIC scoring system (CDSS) (19) are mainly used for the diagnosis of DIC. To facilitate early identification of DIC in sepsis, the ISTH Scientific Standardization Committee proposed a new category termed “sepsis-induced coagulopathy” (SIC) (20). It has been shown that almost all patients meeting ISTH overt DIC also meet the SIC criteria and that SIC always progresses to overt DIC. Therefore, ISTH DIC Scientific Standardization Committee recommends a two-step diagnostic approach to facilitate early recognition of DIC in patients with sepsis, that is, first assessing for SIC and then assessing for ISTH overt DIC after meeting SIC criteria (21).

Following the modification of the Sepsis-3 definition, the population of septic patients changed significantly (2). However, due to the heterogeneity of the sepsis population, conducting large clinical trials has been very difficult and time-consuming. After re-evaluating previous large clinical trials using Sepsis-3 criteria, some *post-hoc* studies have shown that anticoagulation therapy may improve the prognosis of specific populations of sepsis (22, 23). According to our knowledge, there is no consensus on the efficacy of LMWH in patients meeting Sepsis-3 criteria, and no universal scoring systems are available to guide anticoagulant therapy.

Therefore, the present study aimed to reassess the clinical efficacy and safety of LMWH in patients meeting the criteria for Sepsis-3 and further identify the target population that is likely to benefit more from LMWH therapy.

## Materials and methods

### Study design and patients

The patients diagnosed with sepsis were screened from January 2016 to December 2020 in the First Affiliated Hospital of Xi'an Jiaotong University, the largest general hospital in northwest China under the direct administration of the Chinese Ministry of Health. We collected the clinical data retrospectively. The collected data were re-evaluated for fulfilling the Sepsis-3 criteria by two independent reviewers. The critical exclusion criteria were as follows: patients aged younger than 18 years; those with hospital stay time <72 h; those who were pregnant or nursing; those with a high risk for bleeding (including active bleeding, severe traumatic brain injury, cerebral aneurysm, arteriovenous malformation, intracranial surgery within the last 3 months, and history of gastrointestinal bleeding in the last 6 weeks); those showing indications for therapeutic anticoagulation (including acute coronary syndromes, acute venous thromboembolism, and mechanical valves) or ongoing anticoagulation therapy; those having known or suspected heparin allergy or adverse reaction, such as heparin-induced thrombocytopenia; those diagnosed with the hematologic malignant disease; those diagnosed with chronic liver, kidney, heart, or lung insufficiency; recipients of organ transplants; and patients with missing data for the main analysis. We defined the day of a suspected infection combined with an available acute increase in Sequential Organ Failure Assessment (SOFA) score ≥ 2 from baseline as “Day 0”. All patients were given standard medical treatment according to the Surviving Sepsis Campaign Guidelines, which included antimicrobial therapy, fluid therapy, glucose control, supportive care, and nutrition supplements (10). The patients of the study were categorized into the treatment group and the control group based on whether they had received subcutaneous LMWH administration lasting for over 5 days.

The Ethics Committee of The First Affiliated Hospital of Xi'an Jiaotong University waived the need for informed consent because this study used a retrospective and anonymous dataset and approved this study (No. XJTU1AF2022LSK-261).

### Definition of sepsis, septic shock, DIC, and SIC

According to the definition of Sepsis-3 presented at the 45th Critical Care Congress of the Society of Critical Care Medicine in 2016, sepsis is defined as a life-threatening organ dysfunction caused by a dysregulated host response to infection. Septic shock is defined by the presence of hyperlactatemia and sepsis-induced hypotension needing vasopressors after volume resuscitation.

Disseminated intravascular coagulation (DIC) was diagnosed at the time of inclusion based on the ISTH overt DIC criteria (24). The ISTH overt DIC scoring system includes platelet counts, prothrombin time, level of FDP/D-dimer, and fibrinogen content. Since there is no gold standard for the diagnosis of DIC, we also utilized the JAAM DIC criteria and Chinese DIC scoring system to evaluate coagulopathy. SIC was defined as “infection-induced organ dysfunction and coagulopathy”, as proposed by the DIC Scientific Standardization Committee in 2017. The SIC scoring system consists of three items: platelet count, prothrombin time (PT)–international normalized ratio (INR), and the SOFA score. The SOFA score was included to confirm the presence of sepsis, but it did not reflect the severity of sepsis.

### Data collection

The data on baseline characteristics of patients, including demographic information, ICU admission categories, comorbidities, complications, infection sites, severity scores, and laboratory tests, were collected. All data were dynamically recorded on days 0, 3, 7, 14, and 28. SOFA score, Acute Physiology and Chronic Health Evaluation (APACHE) II score, ISTH overt DIC score, JAAM DIC score, CDSS DIC score, and SIC score were calculated based on the values on the day patient met the inclusion criteria.

### Outcome measures

The primary outcome was 28-day all-cause mortality. Secondary outcomes were 90-day mortality, ICU-free days, ventilator-free days, and improvement in inflammation and coagulopathy. ICU-free days is a composite outcome combining mortality and ICU length of stay. The number of ICU-free days was calculated as 28 minus the number of days or part-days spent in the ICU during the first 28 days after enrollment (excluding any days of ICU readmission). Patients who died were assigned the worst possible outcome of zero ICU-free days. A similar approach was applied to assess the number of ventilator-free days. The information regarding bleeding events was also extracted at the same time.

### Statistical analysis

The retrospective design of this study caused baseline imbalances between the treatment and control groups. Therefore, propensity score matching (PSM) was employed to achieve a minimal confounding bias at baseline. In our study, PSM was performed by the nearest neighbor matching using a caliper of 0.02 standard deviations of the estimated propensity score. Patients were matched in a 1:1 ratio. Final covariates included age, sex, APACHE II score, SOFA score, septic shock, ISTH overt DIC, and SIC on admission.

Statistical analysis was performed using SPSS 25.0 for Windows (SPSS, Chicago, IL, USA), with graphs drawn using GraphPad Prism 8.0 (GraphPad, La Jolla, CA, USA). Continuous variables were presented as means ± standard deviation for normal distribution or medians and interquartile range for skewed distribution. Comparisons were analyzed with Student's *t*-test or the non-parametric Mann–Whitney *U*-test, respectively. Categorical data were presented as numbers (percentage) and were compared by Pearson's Chi-square or Fisher's exact test as appropriate. The 28-day survival curves were generated using the Kaplan–Meier method and compared by the log-rank test. In addition, the univariate and stepwise multivariate Cox regression analyses were used to assess the covariates that were associated with 28-day all-cause mortality, and the interactions among variables were tested. For missing variables, predictive mean matching was used to impute numeric features. Statistical significance was set as a *p*-value of <0.05.

## Results

### Baseline characteristics and clinical outcomes of participants

**Figure 1:**
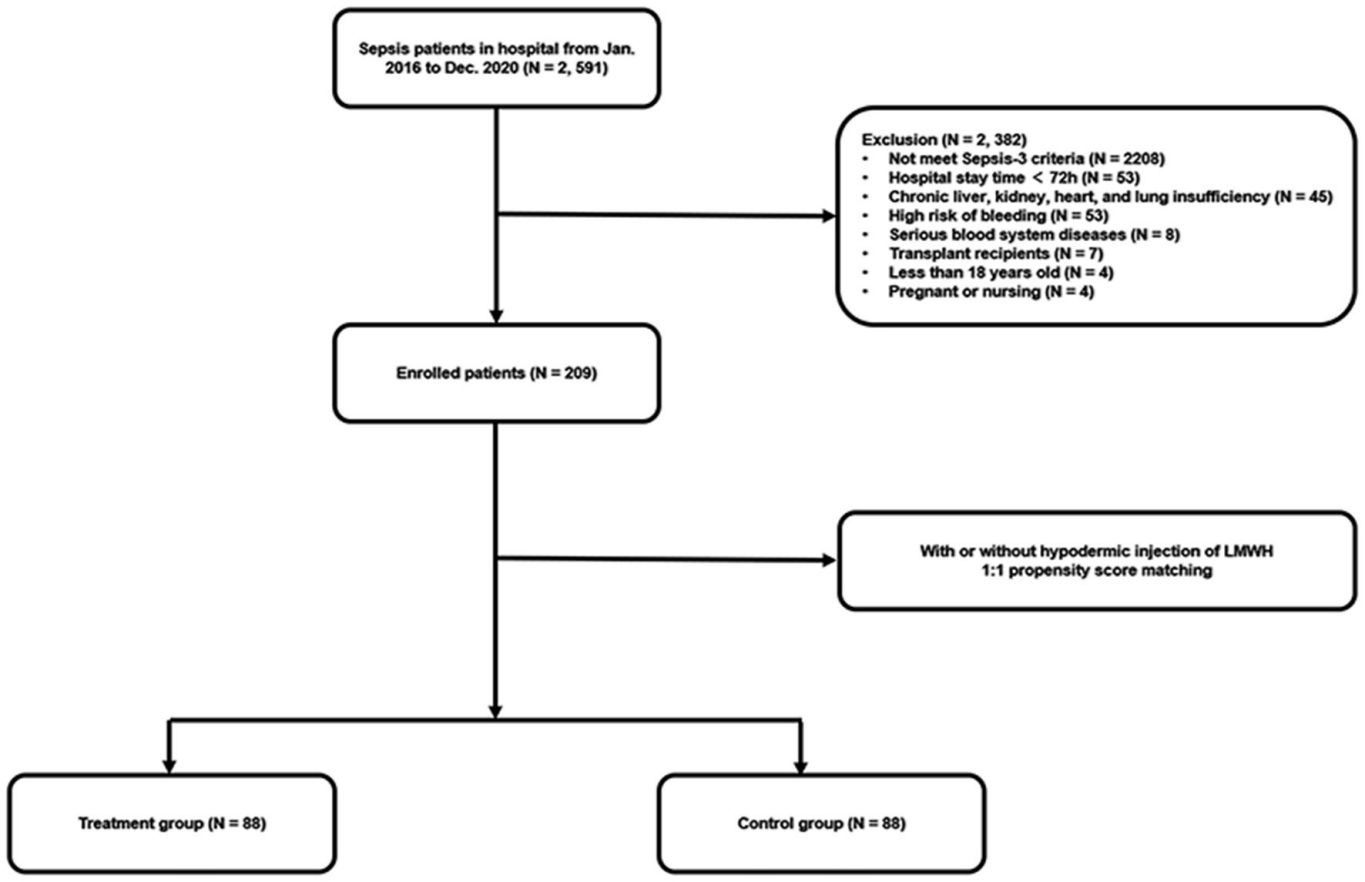
Flow diagram of the population included in the study.

A total of 2,591 septic patients were reviewed from January 2016 to December 2020, as shown in Figure 1. After re-evaluating these patients according to the Sepsis-3 criteria and screening based on exclusion criteria, 209 eligible patients were included in the final cohort. Afterward, 88 pairs of patients were matched in the LMWH group and the control group by 1:1 propensity score matching.

The baseline characteristics of the patients at the time of admission to the ICU and major clinical outcomes were presented in Table 1. After matching, there were no significant differences between the two groups with regard to gender, age, comorbidities, infection sites, severity scores, and complications. The proportion of patients with septic shock was comparable between the two groups (51.1 vs. 52.3%, *p* = 0.880). It should be noted that platelet distribution width (16.50, 16.10–16.90 vs. 16.30, 14.95–16.70, *p* = 0.010), platelet-to-lymphocyte ratio (216.71, 97.81–426.43 vs. 139.15, 70.52–285.96, *p* = 0.040), and systemic immune-inflammation index (SII) (2,315.68, 846.91–3,926.46 vs. 1,160.76, 593.39–2,782.14, *p* = 0.031) were significantly higher in the treatment group than in the control group, which may reflect a higher baseline level of inflammation and endothelial dysfunction in the treatment group.

**Table 1:** Baseline characteristics and clinical outcomes of enrolled patients.

**Variables**	**Treatment group (*N* = 88)**	**Control group (*N* = 88)**	***p*-value**
**Demographics**			
Men, *n* (%)	50 (56.8)	52 (59.1)	0.760
Age, years	64.00 (49.25–74.75)	61.00 (48.00–73.00)	0.719
**ICU admission categories**, ***n*** **(%)**			0.823
MICU	76 (86.4)	77 (87.5)	
SICU	12 (13.6)	11 (12.5)	
**Comorbidities**, ***n*** **(%)**			
Hypertension	34 (38.6)	36 (40.9)	0.758
Diabetes mellitus	26 (29.5)	30 (34.1)	0.517
**Infection sites**, ***n*** **(%)**			
Pneumonia	63 (71.6)	58 (65.9)	0.416
Gastrointestinal	33 (37.5)	36 (40.9)	0.643
Urinary tract infection	10 (11.4)	17 (19.3)	0.143
Bloodstream infection	7 (8.0)	11 (12.5)	0.320
Skin and soft tissue infection	9 (10.2)	9 (10.2)	1.000
Multi-site infection (≥2)	36 (40.9)	39 (44.3)	0.647
**Severity scores**			
SOFA score	9.00 (6.00–12.00)	9.00 (6.00–12.00)	0.811
APACHE II score	19.00 (15.00–26.00)	21.50 (16.00–24.00)	0.708
**Laboratory tests**			
PCT, ng/ml	9.09 (2.53–37.61)	14.68 (4.38–46.75)	0.175
Lac, mmol/L	1.90 (1.00–2.70)	1.90 (1.33–3.75)	0.102
CRP, mg/L	143.85 (75.60–237.20)	139.80 (68.63–226.05)	0.349
Hb, g/L	106.67 ± 27.16	99.10 ± 27.05	0.066
WBC, × 10^9^/L	11.37 (7.50–18.15)	10.57 (7.63–18.64)	0.744
Neutrophil, × 10^9^/L	9.57 (6.78–16.53)	9.20 (6.12–15.86)	0.609
Lymphocyte, × 10^9^/L	0.60 (0.33–0.99)	0.68 (0.33–0.99)	0.756
PDW, fL	16.50 (16.10–16.90)	16.30 (14.95–16.70)	0.010
RDW, fL	45.40 (42.03–49.58)	45.45 (41.75–51.25)	0.556
PLT, × 10^9^/L	115.00 (70.50–171.25)	100.50 (46.00–156.75)	0.097
NLR	17.86 (10.94–30.17)	14.58 (8.27–32.10)	0.552
PLR	216.71 (97.81–426.43)	139.15 (70.52–285.96)	0.040
SII	2,315.68 (846.91–3,926.46)	1,160.76 (593.39–2,782.14)	0.031
APTT, s	45.15 (39.15–53.68)	43.70 (38.08–54.45)	0.585
INR	1.32 (1.16–1.62)	1.36 (1.19–1.62)	0.518
**Laboratory tests**			
FIB, g/L	4.63 (3.07–6.19)	4.39 (3.03–5.74)	0.586
D-dimer, mg/L	5.50 (3.20–12.59)	4.96 (2.46–8.14)	0.071
FDP, mg/L	18.76 (7.80–38.05)	13.17 (6.09–22.35)	0.063
ALT, IU/L	31.00 (20.25–76.00)	31.50 (18.25–61.25)	0.388
TBIL, μmol/L	18.30 (11.78–36.05)	20.70 (12.85–45.10)	0.190
ALB, g/L	27.15 (24.45–30.08)	27.20 (24.88–31.48)	0.403
**Complications**			
Septic shock, *n* (%)	45 (51.1)	46 (52.3)	0.880
SIC, *n* (%)	64 (72.7)	67 (76.1)	0.604
ISTH-DIC, *n* (%)	21 (23.9)	22 (25.0)	0.861
JAAM-DIC, *n* (%)	51 (58.0)	46 (52.3)	0.449
CDSS-DIC, *n* (%)	16 (18.2)	20 (22.7)	0.455
**Clinical outcomes**			
28-day mortality rates, *n* (%)	23 (26.1)	37 (42.0)	0.026
ICU-free days	7.00 (0–18.00)	5.50 (0–19.00)	0.736
Ventilation-free days	22.00 (0–28.00)	23.50 (0–28.00)	0.914

Values reported with mean ± standard deviation (SD), number (percentage), or median (interquartile range). MICU, medical intensive care unit; SICU, surgical intensive care unit; SOFA, sequential organ failure assessment; APACHE, acute physiology and chronic health evaluation; PCT, procalcitonin; Lac, lactic acid; CRP, C-reactive protein; Hb, hemoglobin; WBC, white blood cell; PDW, platelet distribution width; RDW, red blood cell distribution width; PLT, platelet; NLR, neutrophil-to-lymphocyte ratio; PLR, platelet-to-lymphocyte ratio; SII, systemic immune-inflammation index; APTT, activated partial thromboplastin time; INR, international normalized ratio; FIB, fibrinogen; FDP, fibrinogen degradation products; ALT, alanine aminotransferase; TBIL, total bilirubin; ALB, albumin; SIC, sepsis-induced coagulopathy; DIC, disseminated intravascular coagulation; ISTH, International Society on Thrombosis and Haemostasis; JAAM, Japanese Association for Acute Medicine; CDSS, Chinese DIC scoring system.

### Correlation between LMWH treatment and patients' clinical outcomes

**Figure 2:**
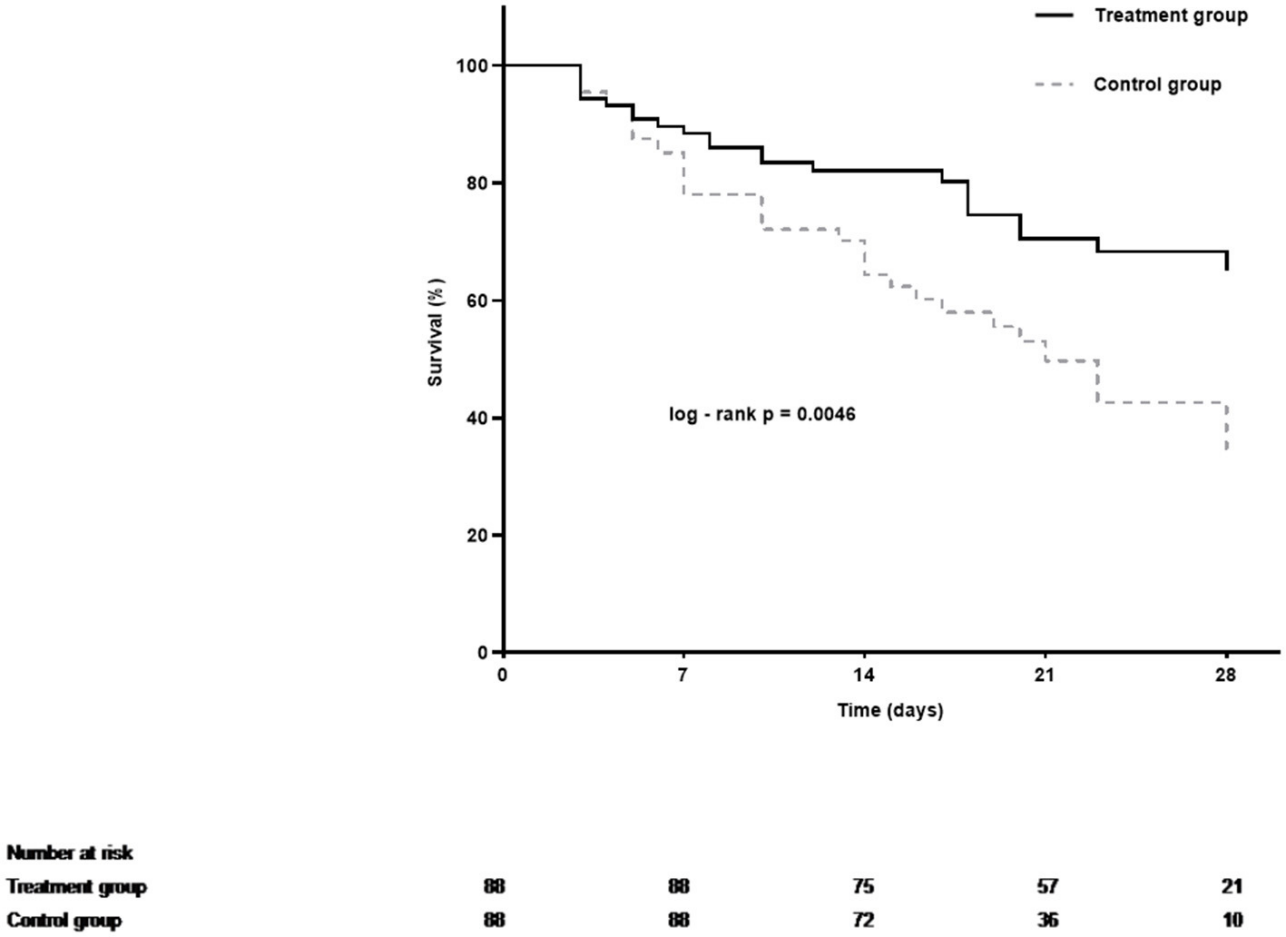
Kaplan–Meier estimates of 28-day cumulative survival probabilities of septic patients in the treatment group and the control group. Treatment with LMWH was associated with a significantly higher rate of survival (Log-rank *p* = 0.0046 by Cox regression analysis).

The overall 28-day mortality rate was found to be 60 out of 176 (34.1%) among the enrolled patients and 44 out of 109 (40.3%) among patients with septic shock on admission. The 28-day mortality rates were 26.1 and 42.0% in the LMWH group and the control group (*p* = 0.026), respectively. Univariate analysis was conducted to determine the indicators associated with 28-day mortality in septic patients (Supplementary Table S1). Cox regression analysis demonstrated that diabetes mellitus (adjusted HR, 2.21; 95% CI, 1.17–4.20; *p* = 0.015), pneumonia (adjusted HR, 2.84; 95% CI, 1.26–6.38; *p* = 0.012), septic shock (adjusted HR, 1.91; 95% CI, 1.03–3.52; *p* = 0.040), and higher APACHE II scores (adjusted HR, 1.05; 95% CI, 1.00–1.09; *p* = 0.037) were independent risk factors for 28-day mortality. More importantly, LMWH administration was the only independent protective factor for sepsis 28-day mortality (adjusted HR, 0.50; 95% CI, 0.29–0.86; *p* = 0.013) (Table 2). Similarly, the 28-day Kaplan–Meier survival curves also revealed that patients in the LMWH group had higher survival probability when compared to those in the control group (HR, 0.49, 95%CI, 0.29–0.81; log-rank *p* = 0.0046) (Figure 2). There were no statistical differences between the two groups in terms of 90-day mortality, ICU-free days, and ventilator-free days.

**Table 2:** Univariate and multivariate analyses of the Cox proportional hazards model for risk of 28-day mortality.

**Variables**	**Univariate analysis**		**Multivariate analysis**	
	**HR (95%CI)**	***p*** **-value**	**Adjusted HR (95%CI)**	***p*** **-value**
Age, years	1.02 (1.01–1.04)	0.011		
Hypertension	1.63 (0.98–2.70)	0.059		
Diabetes Mellitus	1.67 (0.98–2.84)	0.057	2.21 (1.17–4.20)	0.015
Pneumonia	2.79 (1.32–5.87)	0.007	2.84 (1.26–6.38)	0.012
SOFA score	1.11 (1.04–1.19)	0.002		
APACHE II score	1.07 (1.04–1.10)	<0.001	1.05 (1.00–1.09)	0.037
Lac	1.07 (1.01–1.14)	0.029		
INR	1.33 (1.02–1.72)	0.034		
Septic shock	1.88 (1.11–3.20)	0.019	1.91(1.03–3.52)	0.040
SIC	1.31 (0.72–2.40)	0.373		
ISTH-DIC	1.31 (0.79–2.19)	0.301		
JAAM-DIC	0.94 (0.57–1.56)	0.812		
CDSS-DIC	1.11 (0.60–2.05)	0.736		
LMWH	0.48 (0.29–0.81)	0.006	0.50 (0.29–0.86)	0.013

SOFA, sequential organ failure assessment; APACHE, acute physiology and chronic health evaluation; Lac, lactic acid; INR, international normalized ratio; SIC, sepsis-induced coagulopathy; DIC, disseminated intravascular coagulation; ISTH, International Society on Thrombosis and Haemostasis; JAAM, Japanese Association for Acute Medicine; CDSS, Chinese DIC scoring system; LMWH, low-molecular weight heparin.

### Effect of LMWH on inflammation and coagulation disorders

**Figure 3:**
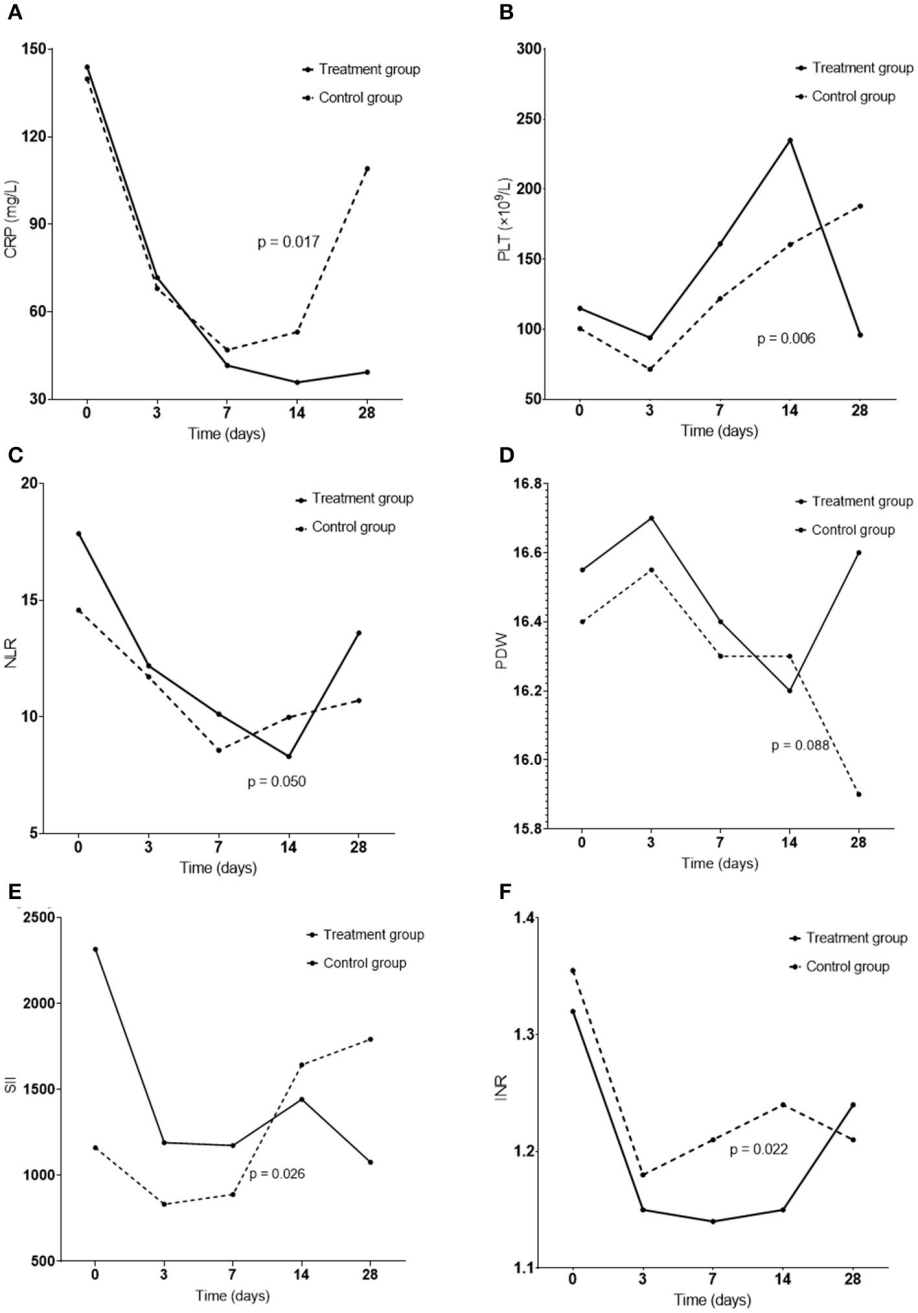
Dynamic changes in inflammation and coagulation indicators including CRP **(A)**, PLT **(B)**, NLR **(C)**, PDW **(D)**, SII **(E)**, and INR **(F)** in the treatment group and the control group in patients with sepsis. The *p*-values were calculated by comparing the changes from days 0 to 14 between the two groups. CRP, C-reactive protein; PLT, platelet; NLR, neutrophil-to-lymphocyte ratio; PDW, red blood cell distribution width; SII, systemic immune-inflammation index; INR, international normalized ratio.

The level of C-reactive protein (CRP), neutrophil-to-lymphocyte ratio (NLR), red blood cell distribution width (PDW), and systemic immune-inflammation index (SII) appeared to decrease with LMWH administration in the first 14 days, indicating reduced inflammatory response (Figure 3). Note that an increase in SII value was displayed in the control group. In addition, dynamic changes in CRP and SII values displayed significant differences between the two groups. Meanwhile, platelet number was increased and the international normalized ratio (INR) was decreased significantly in the treatment group, reflecting improved coagulation, compared to the control group.

### Effect of LMWH treatment in subgroup analysis

**Figure 4:**
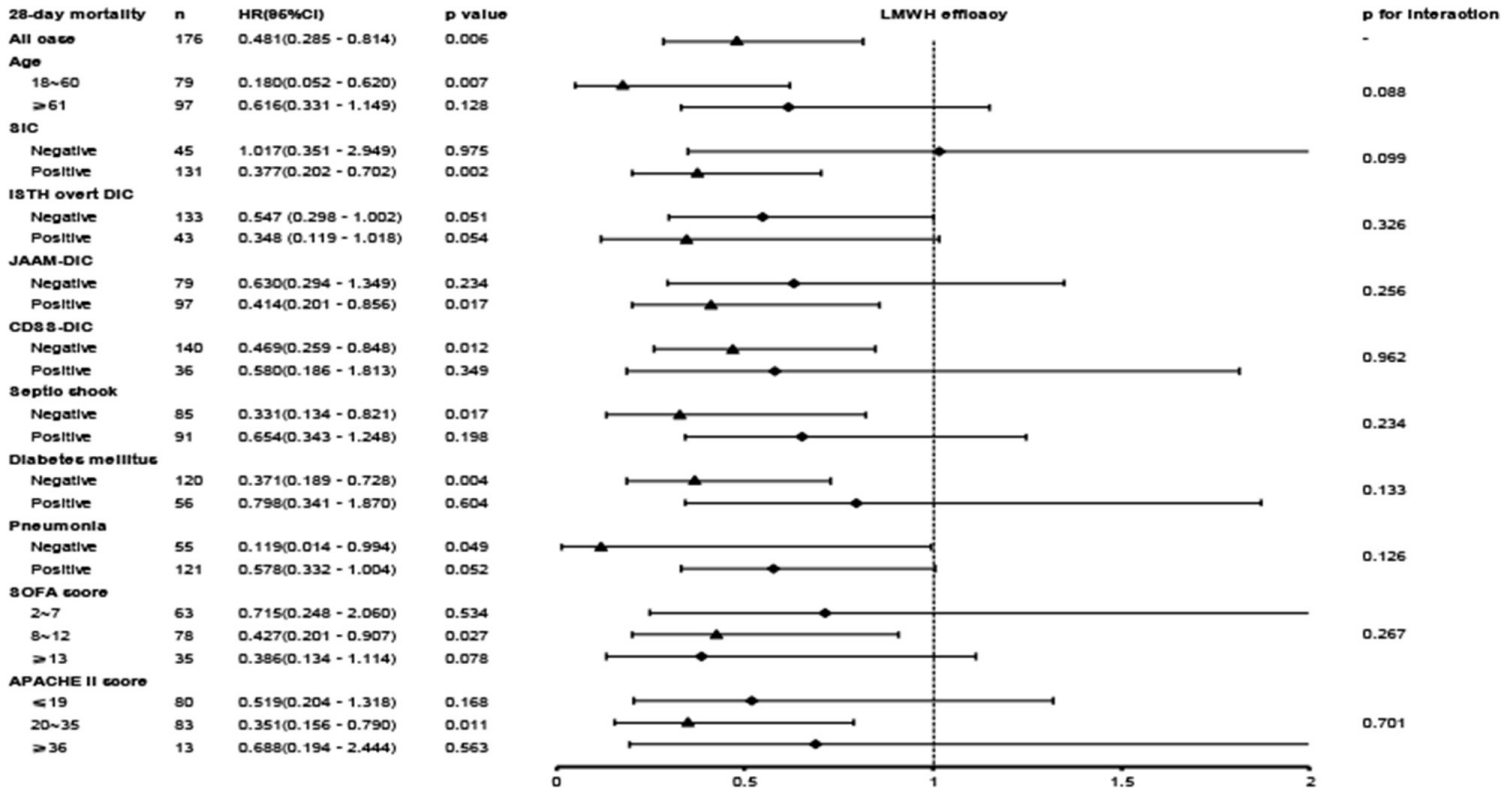
The association between LMWH administration and 28-day mortality in the overall population and subgroups.

A close association between LMWH therapy and the preferable outcome was demonstrated in patients younger than 60 years old and diagnosed with SIC, ISTH overt DIC, non-septic shock, or non-diabetes (Figure 4). When patients were further stratified according to the APACHE II score, an obvious benefit with LMWH treatment was displayed in the moderate-risk group (APACHE II score 20–35), while no significant differences were found in the low-risk subgroup (APACHE II score ≤ 19) and the high-risk subgroup (APACHE II score ≥ 36). The hazard ratio of mortality in the LMWH group was decreased with increasing SOFA score. Similar to the APACHE II score, the SOFA score showed no significant difference in the low-risk subgroup (SOFA score ≤ 7), but a significant reduction in mortality was displayed in the moderate-risk group (SOFA score 8–12). However, a lower HR value (0.386, 95% CI: 0.134–1.114, *p* = 0.078) without significant difference was presented in the patients with higher SOFA score (SOFA score ≥ 13), which was inconsistent with the findings of a previous study (5), while small sample size and high heterogeneity may be attributed to this discrepancy. Further evaluation based on different coagulating score systems showed that septic patients with ISTH overt DIC (HR, 0.319; 95% CI, 0.133–0.770; *p* = 0.011) benefited most from LMWH therapy, followed by SIC (HR, 0.377; 95% CI, 0.202–0.702; *p* = 0.002) and JAAM-DIC (HR, 0.414; 95% CI, 0.201–0.856; *p* = 0.018). Septic patients meeting the CDSS-DIC criteria did not display beneficial effects following LMWH treatment.

### Adverse events

The incidence of bleeding events was similar between the two groups (6.8 vs. 8.0%, *p* = 0.773) (Table 3). No significant difference was found in various bleeding sites. Furthermore, the rates of multi-site bleeding did not show significant differences between the two groups (2.3 vs. 3.4%).

**Table 3:** Bleeding complications in septic patients with or without LMWH treatment.

**Bleeding sites**	**Treatment group (*n* = 88)**	**Control group (*n* = 88)**	***p*-value**
Gastrointestinal bleeding	2 (2.3%)	3 (3.4%)	
Respiratory tract bleeding	2 (2.3%)	0 (0.0%)	
Urinary tract bleeding	0 (0.0%)	1 (1.1%)	
Intracranial bleeding	0 (0.0%)	0 (0.0%)	
Multi-site bleeding (≥2)	2 (2.3%)	3 (3.4%)	
Total	6 (6.8%)	7 (8.0%)	0.773

## Discussion

In the current study, we performed a retrospective investigation to evaluate the effect of LMWH on the prognosis of septic patients meeting Sepsis-3 criteria. Based on propensity score matching, the results demonstrated that LMWH can improve the 28-day mortality in septic patients and also improve inflammation and coagulopathy without increasing the occurrence of bleeding events. LMWH administration was the independent protective factor for septic 28-day mortality. The findings of this study indicate that patients younger than 60 years old and diagnosed with SIC, ISTH overt DIC, non-septic shock, or non-diabetes and patients included in the moderate-risk group (SOFA score, 8–12 and APACHE II score, 20–35) would benefit more from LMWH therapy.

LMWH has been administrated to prevent venous thromboembolism for over 60 years, owing to its rapid and long-lasting antithrombotic effect; however, its efficacy in treating sepsis patients remains controversial. However, with the modification of the Sepsis-3 definition and diagnostic criteria, emphasizing the need for the presence of organ dysfunction, the population of septic patients has changed significantly. Dysregulated host response to infection induces enhanced inflammatory response and coagulopathy, leading to vascular endothelial damage and microvascular thrombosis, which subsequently cause life-threatening organ dysfunction and ultimately lead to sepsis (25). It is widely accepted that inflammation and coagulopathy play an important role in the progression of sepsis. It is worth noting that LMWH, in addition to its well-known anticoagulant effects, exhibits direct and indirect anti-inflammatory properties (26). Fries et al. reported that heparin treatment improved colitis in the rat model (27). Wu et al. showed that LMWH improved the inflammatory state of acute sinusitis rats by inhibiting the TLR4-MyD88-NF-κB signaling pathway (28). Therefore, there is an urgent necessity to reassess the efficacy of LMWH under the Sepsis-3 criteria.

Previous studies have found that adjuvant treatment with LMWH could improve the prognosis of adult septic patients (12, 29), while other studies have demonstrated benefits only in specific populations, such as septic patients with SIC (30) and ISTH overt DIC (22, 30). In the current investigation, we found that LMWH significantly reduced 28-day mortality in adult patients meeting Sepsis-3 criteria. Cox regression analysis showed that LMWH was the only independent protective factor for 28-day mortality in sepsis, and the Kaplan–Meier 28-day survival curve also displayed a higher probability of survival in the LMWH group. Meanwhile, the levels of inflammatory indicators and coagulopathy in the LMWH group were reversed significantly from days 0 to 14 during the course of sepsis. These results positively demonstrated that LMWH therapy provided remarkable protection for patients with Sepsis-3. However, it is notable that, after day 14, the levels of inflammation indicators and coagulopathy began to deteriorate in both groups. The fact that the patients with disease progression remained in ICU for continued observation could be a reason for this deterioration. Furthermore, the development of Persistent Inflammation, Immunosuppression, and Catabolism Syndrome (PICS) and chronic critical illness during ICU treatment may be another explanation for this change (31, 32), which requires further prospective studies to confirm.

Moreover, multivariate regression analysis also identified that diabetes mellitus, pneumonia, septic shock, and higher APACHE II scores on admission were independent risk factors for poor prognosis in sepsis, which is generally consistent with the results of previous investigations (33–35). In consequent subgroup analysis, LMWH treatment also achieved more favorable outcomes in the non-diabetes, non-pneumonia, and non-septic shock groups, illustrating the reliability of these results.

The identification of and appropriate target population for LMWH therapy is a key issue that needs to be addressed. The current study found that LMWH improves the prognosis of all adult patients with Sepsis-3; however, identifying the population that is likely to benefit more from LMWH treatment would be more practical in a clinical setting. Consequently, we conducted a subgroup analysis stratified by factors potentially influencing clinical outcomes. Previous studies have revealed that septic patients with SIC (30), DIC (22, 30, 36–41), and more severe diseases [SOFA score, 13–17 (5) and higher APACHE II score (41)] are more likely to benefit from LMWH treatment. Similarly, our results found that septic patients younger than 60 and diagnosed with SIC, ISTH overt DIC, non-septic shock, or non-diabetes and those included in the “moderate-risk” groups would benefit more from LMWH therapy. Coagulopathy, including SIC and DIC, is a complication and risk factor for sepsis. Hence, it is reasonable that LMWH improves the outcome in septic patients with coagulopathy. We evaluated the ISTH, JAAM, and CDSS DIC scoring systems simultaneously and found that septic patients with ISTH overt DIC could benefit most from LMWH treatment, consistent with the two-step diagnostic approach of SIC and ISTH overt DIC recommended in the guidelines. Additionally, due to “inflamm-aging” and “coagul-aging”, the elderly population is more susceptible to cytokine storm, coagulation dysfunction, and organ dysfunction (42), which influence the efficacy of LMWH in elders.

Further classification of the septic population according to the APACHE II score and SOFA score demonstrated that the benefit of LMWH administration was observed only in the moderate-risk group (SOFA score, 8–12 or APACHE II score, 20–35). On the contrary, previous large clinical trials studying the effects of anticoagulation in septic patients did not find a significant benefit in the overall population, which may be attributed to heterogeneity in the population enrolled, different treatment regimens, and duration of anticoagulation. The KyberSept trial found that high-dose antithrombin III therapy had no effect on 28-day all-cause mortality in adult patients with severe sepsis and septic shock. Similarly, the OPTIMIST trial found that tifacogin treatment had no effect on all-cause mortality in patients with severe sepsis and high INR (14). A randomized, double-blind, placebo-controlled, multicenter trial of drotrecogin alfa (activated) (DrotAA) displayed that DrotAA treatment did not improve 28- and 90-day mortality in patients with septic shock when compared to placebo treatment (15). In the early stage of sepsis, immune thrombosis mediates host protection against pathogens. Hence, anticoagulation is not recommended for patients with a low-risk of sepsis because of a lack of survival benefit and the potential risk of bleeding. As the disease progresses, inflammation persists and thrombosis is activated extensively, leading to coagulopathy or even DIC, which is the optimal time to initiate anticoagulation therapy. However, in the later stage of DIC, the depletion of hemostatic agents increases the risk of bleeding with anticoagulation therapy. These observations indicate that septic patients with moderate severity and in the early stage of DIC could demonstrate survival benefits from LMWH therapy. However, an enlarged sample size is needed to confirm the precise target population, which can be subsequently implemented in clinical practice.

The safety of LMWH therapy is another noteworthy issue. The results of our observation showed that there was no difference in bleeding complications between the LMWH group and the conventional treatment group. Recently, a meta-analysis studying the effect of LMWH also displayed a comparable rate of bleeding complications in the treatment group (12), whereas several meta-analyses showed an increased risk of bleeding with LMWH therapy (43, 44). Therefore, to a certain extent, caution should be exercised when administering LMWH in septic patients, and further high-quality clinical trials are needed to confirm the efficacy and safety of LMWH.

We acknowledge some inevitable limitations in the current study. First, the retrospective nature of this study reduced the strength of the results. Hence, we conducted propensity score matching to minimize baseline confounders. It should be noted that the PDW, PLR, and SII values were still significantly higher in the LMWH group after matching, reflecting the baseline higher platelet activation, elevated host thrombotic/inflammatory response, and poorer prognosis than those observed in the control group (45–50). Nevertheless, current data demonstrated the superiority of LMWH in improving 28-day mortality in sepsis patients. Furthermore, a prospective randomized controlled study in critical patients requires many years to reach the sample size providing statistical power, and ethical issue is another constraint when pursuing randomized controlled clinical trial in such a population. Second, some variables of the patients were not available in this retrospective cohort, such as the dose of LMWH and duration of treatment, which may influence the reliability of the results. Moreover, to identify the optimal target treatment population, we performed a subgroup analysis where potential false-positive results are inevitable. Hence, the results should be interpreted with caution. Additionally, the patients were enrolled in one single medical center. When we conducted the pre-trail estimation, 88 cases in each group were sufficient to evaluate the effect. The statistical power of this study was 0.81, and the results were found to be credible.

## Conclusion

LMWH exhibits a potential life-saving effect in patients meeting Sepsis-3 criteria and alleviates inflammatory response and coagulopathy. Additionally, septic patients diagnosed with SIC or ISTH overt DIC can benefit more from LMWH treatment.

## Data availability statement

The raw data supporting the conclusions of this article will be made available from the corresponding author upon reasonable request.

## Author contributions

ZZ, TY, and DR: study design, study identification, data collection and extraction, data analysis and interpretation, and manuscript drafting. JZ and LL: data collection and quality assessment. JLi, SF, TN, and WX: data analysis and interpretation and critical revision of the manuscript. YY, TC, and YH: study design, interpretation, and critical review of the manuscript. YZ and JLiu: study concept, study design, data analysis, interpretation of data, manuscript revision, quality control of algorithms, and study supervision. All authors contributed equally to the content of the manuscript. All authors contributed to the article and approved the submitted version.
