# Supplementary material for: Low-molecular-weight heparin therapy reduces 28-day mortality in patients with sepsis-3 by improving inflammation and coagulopathy
Source: Front Med (Lausanne). 2023 Jun 9;10:1157775. doi: 10.3389/fmed.2023.1157775 (PMC10289000; doi:10.3389/fmed.2023.1157775)
Supplement: Supplementary file 1 [file Table_1.DOCX]

**TABLE S1:** Univariate analysis for risk of 28-day mortality in patients with sepsis

| **Variables** | **Univariate analysis** | |  | **Variables** | **Univariate analysis** | |
| --- | --- | --- | --- | --- | --- | --- |
|  | **HR (95%CI)** | **P value** |  |  | **HR (95%CI)** | **P value** |
| Male, n (%) | 1.27(0.74-2.15) | 0.385 |  | PDW, fL | 1.04(0.97-1.12) | 0.285 |
| Age, years | 1.02(1.01-1.04) | 0.011 |  | RDW, fL | 1.03(1.00-1.05) | 0.155 |
| MICU/SICU | 0.67(0.30-1.48) | 0.318 |  | PLT, ×10^9^/L | 1.00(1.00-1.00) | 0.887 |
| Hypertension | 1.63(0.98-2.70) | 0.059 |  | NLR | 1.00(0.99-1.01) | 0.847 |
| Diabetes Mellitus | 1.67(0.98-2.84) | 0.057 |  | PLR | 1.00(1.00-1.00) | 0.623 |
| Pneumonia | 2.79(1.32-5.87) | 0.007 |  | SII | 1.00(1.00-1.00) | 0.929 |
| Gastrointestinal | 0.70(0.41-1.19) | 0.184 |  | APTT, s | 1.00(0.99-1.01) | 0.714 |
| Urinary tract infection | 0.46(0.17-1.27) | 0.134 |  | INR | 1.19(0.68-2.09) | 0.546 |
| Bloodstream infection | 0.79(0.32-1.97) | 0.609 |  | FIB, g/L | 1.04(0.91-1.18) | 0.577 |
| Skin and soft tissue infection | 1.05(0.45-2.44) | 0.909 |  | D-dimer, mg/L | 1.00(0.98-1.02) | 0.969 |
| Multi-site infection (≥ 2) | 1.00(0.60-1.66) | 0.989 |  | FDP, mg/L | 1.00(0.99-1.01) | 0.978 |
| SOFA score | 1.11(1.04-1.19) | 0.002 |  | ALT, IU/L | 1.00(1.00-1.00) | 0.694 |
| APACHE II score | 1.07(1.04-1.10) | <0.001 |  | TBIL, μmol/L | 1.00(0.99-1.00) | 0.435 |
| PCT, ng/ml | 1.00(0.99-1.01) | 0.558 |  | ALB, g/L | 1.00(0.96-1.05) | 0.943 |
| Lac, mmol/L | 1.07(1.01-1.14) | 0.029 |  | Septic shock, n (%) | 1.88(1.11-3.20) | 0.019 |
| CRP, mg/L | 1.00(1.00-1.00) | 0.465 |  | SIC, n (%) | 1.31(0.72-2.40) | 0.373 |
| Hb, g/L | 1.00(0.99-1.01) | 0.801 |  | ISTH-DIC, n (%) | 1.15(0.65-2.05) | 0.624 |
| WBC, ×10^9^/L | 0.99(0.96-1.02) | 0.383 |  | JAAM-DIC, n (%) | 0.94(0.57-1.56) | 0.812 |
| Neutrophil, ×10^9^/L | 0.99(0.96-1.02) | 0.463 |  | CDSS-DIC, n (%) | 1.11(0.60-2.05) | 0.736 |
| Lymphocyte, ×10^9^/L | 0.75(0.50-1.12) | 0.160 |  | LMWH | 0.48(0.29-0.81) | 0.006 |
